# Management of bovine brucellosis in organized dairy herds through the identification of risk factors: A cross-sectional study from Karnataka, India

**DOI:** 10.14202/vetworld.2023.1122-1130

**Published:** 2023-05-30

**Authors:** Rajeswari Shome, Krithiga Natesan, Triveni Kalleshamurthy, Chaitra Yadav, Swati Sahay, Somy Skariah, Nagalingam Mohandoss, Obli Rajendran Vinodh Kumar, Bibek Ranjan Shome, Habibur Rahman

**Affiliations:** 1 ICAR-NIVEDI, Bengaluru, Karnataka, India; 2 School of Basic and Applied Sciences, Dayananda Sagar University, Bengaluru, Karnataka, India; 3 Division of Epidemiology, ICAR-Indian Veterinary Research Institute, Bareilly, Uttar Pradesh, India; 4 International Livestock Research Institute, NASC Complex, CG Center, DPS Marg, Pusa, New Delhi, India

**Keywords:** brucellosis, buffalo, cattle, India, risk factors, seroprevalence

## Abstract

**Background and Aim:**

Brucellosis is an infectious disease caused by *Brucella* species. This study aimed to identify the risk factors associated with bovine brucellosis seropositivity in organized dairy farms to control the disease in unvaccinated adult bovine herds in Karnataka, India.

**Materials and Methods:**

In total, 3610 samples (3221 cattle and 389 buffaloes) were subjected to parallel testing using the Rose Bengal plate test and protein G-based enzyme-linked immunosorbent assay, followed by analyses of animal- and farm-level epidemiological datasets to identify the risk factors.

**Results:**

The apparent brucellosis prevalence at the animal level was higher in buffaloes (8.2%, 95% confidence interval [CI] = 5.9–11.4) than in cattle (6.1%, 95% CI = 5.3–7.0). In a multivariable logistic model, animals calved 3–5 times (odds ratio [OR] = 2.22, 95% CI = 1.50–3.1, reference [ref]: animals calved <2 times); animals with a history of abortion (OR = 54.73, 95% CI = 33.66–89.02), repeat breeding (OR = 19.46, 95% CI = 11.72–32.25), and placental retention (OR = 13.94, 95% CI = 4.92–39.42, ref: no clinical signs); and dogs on farms (OR = 2.55, 95% CI = 1.48–4.40, ref: absence of dogs); disposal of aborted fetus in open fields (OR = 4.97, 95% CI = 1.93–12.84) and water bodies (OR = 2.22, 95% CI = 1.50–3.1, ref: buried); purchase of animals from other farms (OR = 6.46, 95% CI = 1.01–41.67, ref: government farms); hand milking (OR = 1.98, 95% CI = 1.02–10.0, ref: machine milking); and use of monthly veterinary services (OR = 3.45, 95% CI = 1.28–9.29, ref: weekly services) were considered significant risk factors for brucellosis in organized bovine herds (p < 0.01).

**Conclusion:**

The study identified that the animals calved 3–5 times or with a history of abortion/repeat breeding/placental retention, and disposal of aborted fetus in open fields/water bodies as the potential risk factors for bovine brucellosis. These risk factors should be controlled through the implementation of best practices to reduce the brucellosis burden in bovine farms.

## Introduction

Brucella species are important zoonotic pathogens infecting livestock, marine mammals, amphibians, and humans. Among the 12 identified species of *Brucella*, *Brucella melitensis*, *Brucella* *suis*, and *Brucella* *abortus* are the most important species exerting detrimental effects on both animal and human health [1]. Brucellosis in bovines is predominantly caused by *B. abortus*, less frequently caused by *B. melitensis*, and occasionally caused by *B. suis* [2]. *Brucella* infection among bovines is characterized by premature abortions and breeding-related complications, such as repeat breeding, retained products of conception, metritis, stillbirths, weakness in offspring, reduced milk production in females, orchitis, and epididymitis in males [3]. Humans are accidental hosts contracting the disease through direct contact with infected animals or indirect contact with contaminated animal products [4]. Approximately 20% of cattle worldwide are infected with *Brucella* [5]. Although substantial control has been achieved for other infectious diseases, brucellosis remains a major public health concern causing massive economic losses attributable to abortions, infertility, and decreased milk production [6].

In India, brucellosis is an endemic disease and control programs involving the vaccination of cattle and buffalo calves with the *B. abortus* S19 vaccine have been implemented in endemic regions since 2012 [7]. Dairy animals are reared in intensive, semi-intensive, extensive, and mixed farming under different agroclimatic conditions. In intensive dairy production, many factors aid the persistence and transmission of brucellosis. A meta-analysis of 39 studies analyzing the seroprevalence of brucellosis in India based on PubMed and IndMed data revealed high rates of brucellosis seropositivity in the states of Karnataka, Maharashtra, Delhi, Kerala, and Kashmir [8]. Comprehensive surveillance, control, and eradication activities are required to reduce brucellosis transmission in these regions, with an appropriate understanding of the associated risk factors [9]. Although a higher prevalence of brucellosis has been reported in Karnataka, which is located in southern India, the risk factors associated with bovine brucellosis in the dairy herds of Karnataka had not been reported before the implementation of control programs.

Hence, the present study aimed to assess the risk factors responsible for the spread of brucellosis in organized dairy herds in Karnataka, which may contribute to the study of brucellosis prevention and control practices in dairy herds.

## Materials and Methods

### Ethical approval and Informed consent

The study was approved by the Institutional Animal Ethics Committee, ICAR- NIVEDI, Bengaluru, India, under the DBT Network Project on Brucellosis/IFD/SAN/3142/2012-13 dated September 27, 2012. The authors have obtained permission from farm owners to publish the data.

### Study period and location

A cross-sectional study was conducted in organized dairy farms from April 2015 to March 2017 to identify the risk factors associated with the seroprevalence of brucellosis. Karnataka state is in the Deccan Plateau of India, bordered by six states and the Arabian Sea to the West. More than 75% of the entire geographical area has arid or semi-arid climate with an average annual rainfall of 1248 mm. The dairy development initiatives/schemes have been very well implemented to provide continuous and regular employment to marginally poor farmers, which resulted in a quantum shift in milk production and currently, Karnataka is the second-highest milk-producing state in the country. During this enormous improvement and dairy intensification, increasing prevalence of brucellosis was alarming to farmers and veterinary healthcare personnel [10].

### Sampling and data collection

**Figure-1:**
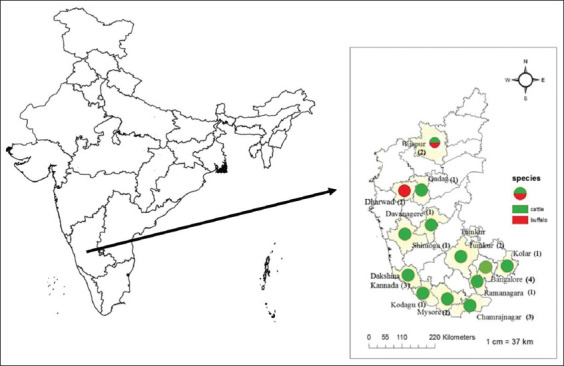
Locations of sample collection in the Karnataka state, India [Source: https://onlinemaps.surveyofindia.gov.in/, The map was constructed using QGIS software version 3.16 (GNU General Public License)].

To retrieve the database of organized dairy farms, the Department of Animal Husbandry, Government of Karnataka, India, was contacted and informed regarding the purpose of this study. Farm owners willing to participate in the study who exclusively maintained either cattle or buffaloes were shortlisted. Twenty-four dairy farms were selected and grouped according to the number of animals (small, medium, and large; Figure-1). For each farm, sample size calculations were performed using a sampling book package in R software with 5–40% prevalence, considering the reported precision of prevalence of 5% at 95% confidence level in Karnataka.

The farm workers and farmers were interviewed regarding farm practices, and animal details were recorded using a close-ended pre-tested questionnaire [11]. Epidemiological data, including species (cattle or buffalo), sex (male or female), cattle breeds (Deoni, Hallikar, Jersey cross, Holstein Friesian, Sahiwal, Gir, or Ongole), buffalo breeds (Murrah or Surti), age (<1, 1–3, 4–6, 7–10, or >10 years), number of calvings (1–2, 3–5, or 6–8), and reproductive history (abortion, repeat breeding, placental retention, stillbirths, pregnancy, and infertility), were collected. The locations of farms were stratified as urban (human settlement with a high population density and infrastructure), periurban (landscape interface in the rural–urban transition zone), and rural (land area located outside towns and cities). Based on the number of animals, the size of farms was classified as small (10–25 animals), medium (25–100 animals), or large (>100 animals) [11]. Rearing methods were categorized as intensive (animals maintained in-house with zero-grazing who were provided feed and water) or semi-intensive (livestock were left free for some time during the day for grazing and maintained in-house for the rest of the day). Other variables included the mode of animal purchase (procured from other farms or governmental agencies); method of milking (machine milking or hand milking); the presence of separate sheds in the farm for sick animals, calves, heifers, and pregnant animals; and type of flooring in the farm (cement or stone flooring). Farm practices regarding the disposal of aborted fetuses (open areas, water bodies, or burial), cleanliness practices and cleaning methods (cleaning with or without disinfectant), frequency of cleaning (once daily or twice weekly), and manure disposal (pit or biogas unit) were recorded. The presence of stray animals, especially dogs, was documented. Regarding healthcare, frequency of veterinary consultation (weekly or monthly), vaccination for brucellosis, and brucellosis awareness were collected to assess their relationships with disease occurrence.

### Serological screening of samples

Approximately 3–5-mL of blood samples were collected from the jugular vein using vacutainer tubes without ethylenediaminetetraacetic acid (Becton Dickinson, UK). Serum was separated from clotted blood after 4–6 h through centrifugation at 5000× *g* for 3–5 min, and separated clear serum was stored at *−*20°C until analysis. The samples were analyzed using the rapid screening Rose Bengal plate test (RBPT) [12]. The colored antigen for RBPT was procured from the Institute of Animal Health and Veterinary Biologicals (Hebbal, Bengaluru, India). The same set of serum samples was analyzed using protein G-based indirect enzyme-linked immunosorbent assay (iELISA), and samples which depict the percentage positivity of <55% and >65% with reference to positive control, were considered as negative and positive, respectively [13].

### Statistical analysis

Data from the questionnaire were digitized into a Microsoft Excel spreadsheet (Microsoft Corporation, Washington, USA). Serological results were interpreted as seronegative (0) or seropositive (1), and datasets were loaded into R version 3.1.1. The apparent prevalence (AP) and true prevalence (TP) of brucellosis were estimated with 95% confidence intervals (CIs) considering the in-house ELISA sensitivities of 98% and 92% and specificity of 95% and 98% for cattle and buffaloes, respectively [13–15]. Odds ratios (ORs) were used to assess the degree of association between potential risk factors and seroprevalence. Seropositivity served as a dependent variable, and risk factors that are likely to predict the outcome variable were considered independent variables. In the first step, associations between independent and dependent variables were examined using Fisher’s exact test. A multivariable logistic regression model was established in the second step using a forward conditional approach based on potential risk factors identified through univariate analysis (factors with p < 0.1). The final model was assessed using the Hosmer-Lemeshow test. In the final multivariable regression model, only factors significant at p < 0.05 were retained.

## Results

In total, 3610 serum samples were obtained from 24 dairy farms. A higher number of samples were obtained from cattle (89.2% [n = 3221]) than from buffaloes (10.8% [n = 389]), and the overall seropositivity rate was 6.3% (228/3610). At the animal level, AP and TP were estimated as 6.1% (95% CI = 5.3–7.0) and 1.2% (95% CI = 0.3–2.1) for cattle and 8.2% (95% CI = 5.9–11.4) and 6.9% (95% CI = 4.3–10.4) for buffaloes, respectively. Among the 24 dairy farms, one farm had an extremely high seroprevalence (28.6%), three farms had a seroprevalence of ≤12.0%, and 13 farms had a seroprevalence of <3% (Table-1).

**Table-1:** Bovine brucellosis seroprevalence in organized dairy farms of Karnataka, India.

Farm	Species	Place of collection	Total no. of animals in the farm	Total samples collected	Total positives	AP (95% CI)	TP (95% CI)
1	Cattle	Tumkur	115	99	3	3.0 (1.0–8.5)	0 (0–3.8)
2	Cattle	Gadag	162	156	17	10.9 (6.9–16.8)	6.3 (0.2–12.6)
3	Cattle	Chamarajanagar	370	355	46	13.0 (9.9–16.9)	8.6 (5.2–12.7)
4	Cattle	Dakshina Kannada	240	225	31	13.8 (9.9–18.9)	9.4 (5.2–15)
5	Cattle	Kodagu	175	162	11	6.8 (3.8–11.7)	1.9 (0–7.3)
6	Cattle	Mysuru	252	225	16	7.1 (4.4–11.2)	2.3 (0–6.8)
7	Cattle	Vijayapura	275	260	17	6.5 (4.1–10.2)	1.7 (0–5.6)
8	Cattle	Mysuru	282	264	3	1.1 (0.4–3.3)	0 (0– 0)
9	Cattle	Bangalore urban	150	141	4	2.8 (1.1–7.1)	0 (0–2.2)
10	Cattle	Davanagere	120	107	2	1.9 (0.5–6.6)	0 (0–1.7)
11	Cattle	Ramanagara	112	94	5	5.3 (2.3–11.9)	0.3 (0–7.4)
12	Cattle	Tumkur	220	202	3	1.5 (0.5–4.3)	0 (0–0)
13	Cattle	Chamarajanagar	256	242	4	1.7 (0.6–4.2)	0 (0–0)
14	Cattle	Bangalore urban	145	133	4	3.0 (1.2–7.5)	0 (0–2.6)
15	Cattle	Dakshina Kannada	174	159	14	8.8 (5.3–14.2)	4.1 (0.34–10.0)
16	Cattle	Shivamogga	65	49	1	2.0 (0.36–10.7)	0 (0–6.1)
17	Cattle	Dakshina Kannada	36	29	0	0 (0–11.7)	0 (0–7.2)
18	Cattle	Chamaraja Nagar	82	70	1	1.4 (0.3–7.7)	0 (0–2.9)
19	Cattle	Kolar	42	34	0	0 (0.0–10.2)	0 (0–5.5)
20	Cattle	Bangalore urban	192	180	4	2.2 (0.9–5.6)	0 (0– 0.6)
21	Cattle	Bangalore urban	45	35	10	28.6 (16.3–45.1)	25.3 (12.2–43.0)
22	Buffalo	Dharwad	161	150	19	12.7 (8.3–18.9)	11.9 (7.0–18.8)
23	Buffalo	Vijayapura	63	58	1	1.7 (0.3–0.91)	0 (0–7.9)
24	Buffalo	Dharwad	192	181	12	6.6 (3.8–11.23)	5.1 (2.0–10.25)

CI=Confidence interval. Apparent prevalence was estimated based on the in-house ELISA sensitivity and specificity, TP=True prevalence, AP=Apparent prevalence

At the animal level, species (buffaloes and cattle), age, and sex were not significantly associated with brucellosis seropositivity. Among the breeds, higher odds of seropositivity (OR = 12.80, 95% CI = 1.82–12.90) were recorded for the indigenous Gir breed of cattle (p < 0.01) and Murrah breed of buffalo (p < 0.05). Animals calved 3–5 times had significantly higher odds of brucellosis seropositivity (OR = 2.53, 95% = CI 1.92–3.33) (p < 0.01). Compared to animals with “no clinical signs” (without any reproductive disorders), higher odds were recorded for animals with a history of abortion (OR = 38.98, 95% CI = 26.50–52.20), repeat breeding (OR = 18.40, 95% CI = 12.10–7.90), and placental retention (OR = 7.34, 95% CI = 2.97–18.21). Compared with animals in urban farms, those in rural farms had significantly higher odds of seropositivity (OR = 1.62, 95% CI = 1.13–2.32), whereas those in periurban farms had lower odds of seropositivity (OR = 0.62, 95% CI = 0.38–1.00; Tables-2 and 3).

**Table-2:** Bivariate analysis of animal and farm-level risk factors for bovine brucellosis in organized dairy herds.

S. No.	Risk factors	No. of samples, n = 3610, (%)	Seropositives, n = 228, (%)	Risk specific seroprevalence (%)	p-value^a^	Odds ratio^a,b^ (95% CI)
1	Species^#^					
	Buffalo	389 (10.78)	32 (14.04)	8.23	0.10	1.38 (0.94–2.04)
	Cattle	3221 (89.22)	196 (85.96)	6.09		1 (Ref)
2	Sex^#^					
	Female	3447 (95.48)	219 (96.05)	6.35	0.67	1.16 (0.58–2.30)
	Male	163 (4.51)	9 (3.94)	5.52		1 (Ref)
3	Breed^#^ (Buffalo)					
	Murrah	331 (85.08)	31 (13.59)	9.37	0.05	0.18 (0.02–1.12)
	Surti	58 (14.91)	1 (0.43)	1.72		1 (Ref)
4	Method of rearing^@^					
	Intensive	3135 (86.84)	205 (89.91)	6.54	0.16	1.36 (0.88–2.14)
	Semi-intensive	475 (13.16)	23 (10.09)	4.84		1 (Ref)
5	Milking method^@^					
	Hand milking	1345 (37.26)	97 (42.54)	7.21	0.09	1.26 (0.96–1.66)
	Machine milking	2265 (62.74)	131 (57.46)	5.78		
6	Method of cleaning^@^					
	Water with disinfectant	1944 (53.85)	92 (40.35)	4.73	0.01	1.78 (1.36–2.35)
	Only with water	1666 (46.15)	136 (59.65)	8.16		1 (Ref)
7	Frequency of cleaning sheds^@^					
	Twice a week	1611 (44.63)	119 (52.19)	7.39	0.02	0.73 (0.57–0.97)
	Once a day	1999 (55.37)	109 (47.81)	5.45		1 (Ref)
8	Manure disposal^@^					
	Pit	2994 (82.94)	200 (87.72)	6.68	0.05	1.50 (1.01–2.25)
	Biogas	616 (17.06)	28 (12.28)	4.55		1 (Ref)
9	Separate sheds for sick, calves, heifer, pregnant^@^					
	Yes	3540 (98.06)	225 (98.68)	6.36	0.48	1.52 (0.47–4.85)
	No	70 (1.94)	3 (1.32)	4.29		1 (Ref)
10	Flooring in the shed^@^					
	Stone flooring	1822 (50.47)	173 (75.88)	9.50	0.01	0.32 (0.19–0.44)
	Cement flooring	1788 (49.53)	55 (24.12)	3.08		1 (Ref)
11	Dogs in the farm^@^					
	Yes	1430 (39.61)	105 (46.05)	7.34	0.04	1.33 (1.01–1.67)
	No	2180 (60.39)	123 (53.95)	5.64		1 (Ref)
12	Frequency of veterinary services^@^					
	Monthly	3053 (84.57)	211 (92.54)	6.91	0.01	2.35 (1.42–3.89)
	Weekly	557 (15.43)	17 (7.46)	3.05		1 (Ref)
13	Vaccination for brucellosis^@^					
	Yes	1300 (36.01)	69 (30.26)	5.31	0.06	1.3 (0.96–1.74)
	No	2310 (63.99)	159 (69.74)	6.88		1 (Ref)
14	Brucellosis awareness^@^					
	No	3117 (86.34)	206 (90.35)	6.61	0.07	0.66 (0.42–1.04)
	Yes	493 (13.66)	22 (9.65)	4.46		1 (Ref)

a Number of seropositive were used for estimation of p-value and odds ratio, ^b^Fisher’s exact test,

# Animal level factors,

@ Farm-level factors

**Table-3:** Binary logistic regression of animal and farm-level risk factors for bovine brucellosis in organized dairy herds.

S. No.	Risk factors	No. of samples, n = 3610, (%)	Seropositive, n = 228, (%)	Risk specific seroprevalence (%)	p-value	Odds ratio (95% CI)
1	Age^#^					
	<1 year	26 (0.72)	0 (0.00)	0.00	0.50	NC
	1–3 year	2300 (63.71)	111 (48.68)	4.83	0.68	0.84 (0.22–3.60)
	4–6 year	814 (22.54)	88 (38.59)	10.81	0.57	2.00 (0.51–8.06)
	7–10 year	435 (12.04)	27 (11.84)	6.21	0.92	1.10 (0.27–4.80)
	>10 year	35 (0.96)	2 (0.87)	5.71	0.88	1 (Ref)
2	Breeds^#^ (Cattle)					
	HF cross	1955 (60.69)	147 (64.47)	7.52	0.72	2.4 0 (0.42–24.85)
	Jersy cross	712 (22.10)	24 (10.52)	3.37	0.99	1.12 (0.19–11.89)
	Deoni	238 (7.38)	7 (3.07)	2.94	0.99	0.97 (0.16–142.90)
	Gir	35 (1.08)	10 (4.38)	28.57	<0.01	12.80 (1.82–12.90)
	Hallikar	53 (1.64)	2 (0.87)	3.77	0.99	1.25 (0.14–18.70)
	Ongole	142 (4.40)	0 (0.00)	0.00	0.18	NC
	Sahiwal	33 (1.02)	1 (0.00)	3.03		1 (Ref)
3	No. of calvings^#^					
	6–8	192 (5.31)	6 (2.63)	3.13	0.33	0.66 (0.28–1.52)
	3–5	991 (27.45)	109 (47.80)	11.00	<0.01	2.53 (1.92–3.33)
	0–2	2427 (67.22)	113 (49.56)	4.66		1 (Ref)
4	History of the animal^#^					
	Abortions	146 (4.04)	79 (34.64)	54.11	<0.01	38.98 (26.50–52.20)
	Repeat breeding	126 (3.49)	45 (19.73)	35.71	<0.01	18.40 (12.10–27.90)
	Retention of placenta	33 (0.91)	6 (2.63)	18.18	<0.01	7.34 (2.97–18.21)
	Still births	31 (0.85)	2 (0.87)	6.45	0.26	2.28 (0.54–9.70)
	Pregnant	4 (0.11)	0 (0.00)	0.00	0.60	NC
	No clinical signs	3270 (90.58)	96 (42.10)	2.94		1 (Ref)
5	Location of the farm^@^					
	Peri–urban	918 (25.43)	30 (13.16)	3.27	0.05	0.62 (0.38–1.00)
	Urban	749 (20.75)	39 (17.11)	5.21		Ref
	Rural	1943 (53.82)	159 (69.74)	8.18	0.05	1.62 (1.13–2.32)
6	No. of animals in the farm^@^					
	Small farm	12 (0.33)	2 (0.88)	16.67	0.14	2.90 (0.63–12.20)
	Medium farm	463 (12.83)	21 (9.21)	4.54	0.09	0.67 (0.42–1.06)
	Large farm	3135 (86.84)	205 (89.91)	6.54		1 (Ref)
7	Mode of procurement of animals^@^					
	Own raised	412 (11.41)	20 (8.77)	4.85	0.16	2.30 (0.70–8.19)
	Procured from Other farms	3054 (84.60)	205 (89.91)	6.71	0.03	3.38 (1.07–8.20)
	Procured from Govt. agencies	144 (3.99)	3 (1.32)	2.08		1 (Ref)
8	Disposal of aborted materials/fetus^@^					
	Open discard	1348 (37.34)	64 (28.07)	4.75	<0.01	1.70 (1.26–2.31)
	Disposed in water bodies	455 (12.60)	40 (17.54)	8.79	<0.01	2.50 (1.70–3.60)
	Buried	1807 (50.06)	124 (54.39)	6.86		1 (Ref)

Small farm-10–25 animals, medium farm- 25–100 anima, large farm- >100 animals, Repeat breeders: Animals with normal estrous cycle without any abnormalities that failed to conceive after 3 or more successful insemination, Still birth: Expulsion of pre-term dead fetus, No clinical signs: without any reproductive disorder clinical signs,

# Animal level factors,

@ Farm-level factors, NC=Not calculated, CI: confidence interval, Hosmer-Lemeshow test Chi-square value is 04.40, p *=* 0.42

At the farm-level, the rearing system, use of separate sheds (for sick animals, calves, heifers, and pregnant animals), and size of the farms (small, medium, and large) were not significantly associated with brucellosis seropositivity. However, significantly higher odds of brucellosis were observed in farms practicing cleaning with only water (OR = 1.78, 95% CI = 1.36–2.35) than farms practicing cleaning with disinfectants. Farms availing monthly veterinary services (OR = 2.35, 95% CI = 1.42–3.89) had higher odds of brucellosis seropositivity than those availing weekly services, and farms practicing disposal of aborted fetuses in water bodies (OR = 2.50, 95% CI = 1.70–3.60) and open fields (OR = 1.70, 95% CI = 1.26–2.31) had higher odds of brucellosis seropositivity than those practicing burial of aborted fetuses (p < 0.01). Greater odds of brucellosis seropositivity (p < 0.05) were recorded for farms that procured animals from other farms (OR = 3.38, 95% CI = 1.07–8.20) than for those that procured animals from government agencies, farms that kept dogs (OR = 1.33, 95% CI = 1.01–1.67), farms that practiced disposal of manure in pits around sheds (OR = 1.50, 95% CI = 1.01–2.25), and farms that cleaned the sheds twice weekly (OR = 1.38, 95% CI = 1.05–1.80). Further, farms with cement flooring had lower odds of brucellosis seropositivity than those with stone flooring (OR = 0.32, 95% CI = 0.19–0.44). However, vaccination against brucellosis and lack of brucellosis awareness among farmers was not considered significant risk factors for brucellosis seropositivity in the bivariate regression analysis (Table-3).

In the multivariable logistic model, animals calved 3–5 times (OR = 2.22, 95% CI = 1.50–3.1, reference [ref]: animals calved <2 times); animals with a history of abortion (OR = 54.73, 95% CI = 33.66–89.02), repeat breeding (OR = 19.46, 95% CI = 11.72–32.25), and placental retention (OR = 13.94, 95% CI = 4.92–39.42, ref: clinical signs); presence of dogs in farms (OR = 2.55, 95% CI = 1.48–4.40, ref: absence of dogs); disposal of aborted fetuses in open fields (OR = 4.97, 95% CI = 1.93–12.84) or water bodies (OR = 2.22, 95% CI = 1.50–3.1); purchase of animals from other farms (OR = 6.46, 95% CI = 1.01–41.67, ref: government agencies); use of hand milking (OR = 1.98, 95% CI = 1.02–10.0, ref: machine milking); monthly veterinary services (OR = 3.45, 95% CI = 1.28–9.29, ref: weekly services), and brucellosis awareness among farm personnel (OR = 0.04, 95% CI = 0.01–0.17) were identified as significant risk factors for brucellosis in organized bovine herds (p < 0.01, Table-4).

**Table-4:** Multivariable analysis of risk factors for bovine brucellosis in organized dairy herds.

Factors	Odds ratio	p-value	Lower 95% CI	Upper 95% CI
No. of calvings				
0–2	Ref			
3–5	2.22	0.01	1.50	3.10
6–8	0.98	0.97	0.38	2.55
Farm location				
Urban	Ref			
Semi urban	0.03	0.01	0.01	0.08
Rural	0.29	0.01	0.12	0.68
Farm size				
Small	NC			
Medium	0.92	0.83	0.01	38.16
Large	Ref			
Manure disposal				
Pit	1.39	0.50	0.53	3.69
Bio-gas	Ref			
Flooring				
Stone flooring	Ref			
Cement flooring	0.40	0.06	0.15	1.04
Dogs in farm				
Presence of stray dogs	2.55	0.01	1.48	4.40
Absence of stray dogs	Ref			
Disposal of aborted materials/fetus				
Open discard	4.97	0.01	1.93	12.84
Disposed in water bodies	33.27	0.01	9.0	123.22
Buried	Ref			
History of the animal				
Abortions	54.73	0.01	33.66	89.02
Repeat breeding	19.46	0.01	11.72	32.25
Retention of placenta	13.94	0.01	4.92	39.42
Still births	4.42	0.06	0.95	20.60
Pregnant	0.00	0.99	0.00	0.00
No clinical signs	Ref			
Mode of procurement of the animals				
Own raised	1.43	0.72	0.21	10.03
Procured from other farms	6.46	0.05	1.01	41.67
Procured from Govt. agencies	Ref			
Milking method				
Hand	1.98	0.04	1.02	3.8
Machine	Ref			
Method of cleaning				
Only water	1.65	0.13	0.86	3.16
Water with disinfectant	Ref			
Frequency of cleaning sheds				
Once in a day	0.52	0.02	0.29	0.91
Twice a week	Ref			
Frequency of veterinary services obtained				
Weekly	Ref			
Monthly	3.45	0.01	1.28	9.29
Vaccination for brucellosis				
Yes	Ref			
No	1.61	0.16	0.82	3.16
Brucellosis awareness				
Yes	0.04	0.01	0.01	0.17
No	Ref			

Constant in the model: −4.647, p *<* 0.001, CI=Confidence interval, Hosmer-Lemeshow test Chi-square value is 11.21, p *=* 0.122, NC=Not calculated

## Discussion

Cattle and buffalo populations in India rank first and second worldwide, respectively, and are the livelihood assets of rural households. Many productive, reproductive, and health-related challenges are emerging in the dairy sector, and bovine brucellosis is a major concern in the intensive dairy production system [13]. With the absence of test and slaughter policies in most Indian states, vaccination and management measures must be implemented to control the risk factors contributing to the re-emergence of brucellosis. In this study, 24 dairy farms were investigated, and most samples were obtained from cattle (89.2% [n = 3221]) as the cattle population is considerably larger (9.16 million) than the buffalo population (3.28 million) in Karnataka [16]. In a previous study, two commonly used tests (RBPT and iELISA) were applied simultaneously to maximize the accuracy of the results because RBPT is known to detect immunoglobulin (Ig)G1 and IgM produced during the acute phase, whereas iELISA is known to predominantly detect IgG in chronic cases of brucellosis [17]. However, RBPT, buffered plate agglutination test, complement fixation test, ELISA, and fluorescent polarization assay are recommended for brucellosis screening in herds and individual animals [1]. In this study, the combined results of RBPT and iELISA were used for risk analysis.

Among 24 dairy farms, the highest seroprevalence was recorded as 28.6% at one farm, followed by 12.0% at three farms, whereas 13 farms had a seroprevalence of <3%. In a previous study, the seroprevalence of bovine brucellosis ranging from as low as 0.7% in unorganized farms to as high as 6.6% in organized herds reflects farm-to-farm variations in brucellosis seroprevalence in Karnataka [18]. At the species level, brucellosis seroprevalence was non-significantly higher in buffaloes (8.2%) than in cattle (6.09%), in line with results from North India, as buffalo is the dominant species in this region [19]. In the present study, age was not a significant factor associated with brucellosis risk, which is contradictory to prior findings [20]. Further, males and females were equally susceptible to brucellosis [21]. However, the potential spread of the disease by infected adult male cattle through infected semen is important in natural breeding practiced mainly by indigenous breeds.

Regarding breed predisposition, the indigenous Gir breed of cattle and Murrah breed of buffalo were more susceptible to brucellosis, which was also reported in another study [22]. Animals calved 3–5 times had significantly higher odds of brucellosis seropositivity (p < 0.01), which was attributed to latent infection or overt clinical manifestations in pregnant adult animals caused by *Brucella* [23]. Brucellosis seropositivity was recorded in animals with histories of abortion, repeat breeding, and placental retention, consistent with prior findings [19, 21].

The rearing system (semi-intensive and intensive), type of milking, use of separate sheds (for sick animals, calves, heifers, and pregnant animals), and size of the farm had no associations with brucellosis seropositivity. Based on this finding, it is evident that larger farms that opt for machine milking can safely use machines after milking healthy animals and before milking animals with brucellosis [24]. Good hygiene is a protective factor for brucellosis [25], whereas unhygienic practices facilitate the spread of infection [26]. In Karnataka, storing dung in a pit away from the shed for a short period before use as fertilizer or for biogas production is a usual practice in many farms. The infected discharges mixed with dung tend to remain in and around the farm for several days, which could lead to soil, water, and feed contamination. Dairy shed floors are usually built with stone or cement. Stone flooring is usually uneven with a rough surface, making it difficult to maintain good hygiene. Animals maintained in sheds with stone flooring had significantly higher rates of brucellosis seropositivity (p < 0.01), which is mainly attributable to the difficulty in maintaining cleanliness. This finding provides important information for farmers starting new dairy ventures.

Aborted material is the main source of disease transmission because healthy animals encounter infected materials and discharge directly or indirectly through food and water. Hence, appropriate handling and disposal of aborted material are essential to prevent the spread of disease to animals and humans on the farms [24]. Exposure to contaminated material and poor management practices are linked to higher seropositivity rates on farms [27]. Unrestricted animal movement, introduction of new animals into farms, and frequent animal purchases for farm replacement or breeding are considered important risk factors for brucellosis [28]. Most of the herds (60.39%) were located on farms with dogs. Aborted fetuses, cotyledons, placental tissues, and viscous discharges are carried away or eaten by dogs on dairy farms. Dogs infected with *B. abortus*, in turn infect cattle [29], and poor biosecurity measures, such as lack of control of visitors and stray animals, contribute to the high prevalence of brucellosis [30]. The use of monthly veterinary services and lack of brucellosis awareness among farmers were identified as the most significant risk factors for brucellosis in organized bovine herds. High brucellosis seropositivity rates have been reported in farms that cannot avail veterinary services [31]. Similarly, <5% of farmers are aware of the potential of brucellosis to spread zoonotically from cattle to humans [32] and from livestock to humans and wildlife reservoirs [33].

In a multivariable logistic model, calving 3–5 times; urban or rural location; histories of abortion, repeat breeding, and placental retention among the animals; the purchase of animals from other farms; the presence of dogs on farms; disposal of aborted fetuses in open fields or water bodies; and hand milking were significantly associated (p < 0.01) with brucellosis. Farm practices such as the use of monthly veterinary services, disposal of manure in pits, and stone flooring were significant risk factors for brucellosis seropositivity. Similar to the present findings, brucellosis was significantly more common in organized farms, crossbred animals, and animals with histories of abortion and repeat breeding than in apparently healthy animals [34]. The herd and individual risk factors associated with bovine brucellosis in Haryana and Punjab [11] and risk factors in periurban areas under intensive production systems in Gujarat have been reported. However, in the present study, both animal- and farm-level risk factors were analyzed and compared among urban, periurban, and rural areas in Karnataka. The study recorded higher brucellosis prevalence in buffaloes than in cattle, and eight animal- and farm-level risk factors significantly influenced the risk of brucellosis in farms (p < 0.01). Implementing practices to control these risk factors can reduce the brucellosis burden in bovine farms.

In a prior study, veterinarians ranked foot and mouth disease and brucellosis first and fourth, respectively, in the list of 10 diseases with economic impacts on the country [14]. Despite its high ranking, brucellosis’s indirect economic impact and zoonotic implications remain undermined. Hence, mass vaccination, environmental hygiene, and personal protection have been emphasized to attain a brucellosis-free equilibrium in cattle for >6 years [35, 36]. Strengthened information dissemination, improved veterinary and public health surveillance, and establishment of diagnostic facilities may add value to the disease control program [37]. Some of the identified animal- and farm-level risk factors can be easily mitigated through awareness programs, which will ultimately strengthen ongoing efforts and vaccination policies for young animals.

## Conclusion

Tests and slaughter are prohibited in India; hence, vaccination and management measures are needed to mitigate the risks associated with the re-emergence of brucellosis. Management measures include defining the type of rearing, improving husbandry practices, increasing brucellosis awareness among farmers, and prioritizing brucellosis control through vaccination. Vaccination with S19 has contributed enormously to the success of many control programs and a reduction in the number of brucellosis cases in humans [38]. In India, compulsory long-term vaccination strategies and implementation of risk-based control measures are needed to reduce the prevalence of brucellosis.

## Authors’ Contributions

RS: Conceptualization and writing-review and editing. KN, CY, ORVK, SS, and SoS: Methodology. TK and NM: Data collection and analysis. HR, RS, BRS, and NM: Supervision of the study. RS and TK: Writing-original draft. All authors have read, reviewed, and approved the final manuscript.
